# Mitochondria-Localized Glutamic Acid-Rich Protein (MGARP) Gene Transcription Is Regulated by Sp1

**DOI:** 10.1371/journal.pone.0050053

**Published:** 2012-11-27

**Authors:** Da Jin, Rui Li, Dongxue Mao, Nan Luo, Yifeng Wang, Shaoyong Chen, Shuping Zhang

**Affiliations:** 1 State Key Laboratory of Biomembrane and Membrane Biotechnology, School of Life Sciences, Tsinghua University, Beijing, China; 2 Beth Israel Deaconess Medical Center, Harvard Medical School, Boston, Massachusetts, United States of America; Baylor College of Medicine, United States of America

## Abstract

**Background:**

Mitochondria-localized glutamic acid-rich protein (MGARP) is a novel mitochondrial transmembrane protein expressed mainly in steroidogenic tissues and in the visual system. Previous studies showed that MGARP functions in hormone biosynthesis and its expression is modulated by the HPG axis.

**Methodology/Principal Findings:**

By bioinformatics, we identified two characteristic GC-rich motifs that are located proximal to the transcription start site (TSS) of MGARP, and each contains two Specificity protein 1 (Sp1) binding elements. We then determined that the −3 kb proximal MGARP promoter is activated in a Sp1-dependent manner using reporter assays and knockdown of Sp1 led to decreased expression of endogenous MGARP messages. We also demonstrated that one of the two GC-rich motifs, GC-Box1, harbors prominent promoter activity mediated by Sp1, and that it requires both GC boxes for full transcriptional activation. These findings suggest a dominant role for these GC boxes and Sp1 in activating the MGARP promoter through a synergistic mechanism. Consistently, the results of an Electrophoretic Mobility Gel Shift Assay (EMSA) and Chromatin Immunoprecipitation (ChIP) confirmed that Sp1 specifically interacts with the GC-rich region. We further found that estrogen receptor α (ERα), a known Sp1 co-activator, could potentiate GC-boxes containing MGARP promoter activity and this effect is mediated by Sp1. Knockdown of Sp1 significantly diminished the MGARP promoter transactivation and the expression of endogenous MGARP mediated by both Sp1 and ERα.

**Conclusions/Significance:**

The present study identified a proximal core sequence in the MGARP promoter that is composed of two enriched Sp1 binding motifs and established Sp1 as one major MGARP transactivator whose functions are synergistic with ERα, providing a novel understanding of the mechanisms of MGARP gene transcriptional regulation.

## Introduction

Mitochondria-localized glutamic acid-rich protein (MGARP) was first identified in the ovary as the ovary-specific acidic protein (OSAP) [1] and, thereafter, it was identified in the cornea [2] and retina [3]. Since this protein is made up of abundant glutamic acids and has specific mitochondrion localization, it was given a universal name, MGARP [3]. Our previous studies have demonstrated that MGARP is highly expressed in the inner segment of the photoreceptor (IS), outer plexiform layer (OPL) and ganglion cell layer (GCL) of the retina, which are enriched with mitochondria [3]. Additional studies have indicated that MGARP is involved in steroidogenesis through its ability to maintain mitochondrial abundance and morphology, and importantly, it is also highly expressed in the organs involved in steroidogenesis, such as the ovary, testis, adrenal gland and brain [4], [5]. MGARP can also be induced by HIF-1 and hypoxia, biasing mitochondrial transport in the anterograde direction and joining the mitochondrial dance [6], [7]. Our recent study reported temporal and tissue-specific expression patterns of MGARP during mouse development [5]. The MGARP protein cannot be detected in the ovary or testis until 2–4 weeks after birth, likely depending on the availability of particular steroids [5]. Furthermore, MGARP expression correlates with estrogen levels in the ovaries during the estrous cycle and it can be up-regulated by estrogen and down-regulated by a GnRH antagonist through a feedback regulatory mechanism [5].

Steroid hormones play pivotal functions in the animal body throughout life. Their major physiological functions include the regulation of behavior, mood, reproduction, development, sex differences in brain function, aging, responses to the environmental stimuli and development of various diseases [8]–[11]. The activity of steroid hormones is mediated by specific effectors such as steroid receptors that function as ligand-activated transcription factors [12], [13]. Estrogens can bind to the estrogen receptor (ER) and stimulate its translocation into the nucleus, where ERs bind to chromatin via specific ER-regulated elements (ERE) to activate downstream gene transcription [14], [15]. It is also known that transactivators, including steroid receptors and particularly ER, depend on co-factors (co-activators versus co-repressors) for full transcriptional regulation [16], [17]. Meanwhile, ER also serves as a co-factor for other transactivators [18].

As a well established general transcriptional factor, Sp1 interacts with GC or GT boxes on the DNA backbone via its highly homologous zinc-finger domain [18], [19]. Its N-terminal glutamine- and serine/threonine-rich domain can function as a transactivator, and its C-terminus has a synergistic activation function through its interaction with other transcription factors [20]. Sp1 is implicated in a variety of biological processes, such as early embryonic development, the G1 phase of the cell cycle, and importantly, steroid receptor-mediated transcription [19], [21], [22]. Sp1 can interact with ERα and contribute to transcriptional outcomes [15], [23]–[26].

As mentioned above, reports have documented that MGARP participates in steroid synthesis, and steroids also regulate MGARP expression [4], [5]. However, the detailed regulatory mechanisms of MGARP gene expression remain unknown. In the present study, we have carried out a characterization study of the MGARP promoter. Using bioinformatics, we identify two classic Sp1-binding GC-rich motifs (−150 bp/−40 bp and −39 bp/0 bp) proximal to the transcription start site (TSS). We demonstrate that reporters driven by the MGARP promoters containing the specific GC-rich motifs are activated by Sp1, and are shown by EMSA and ChIP to also bind Sp1. We also determine that ERα could further enhance the activity of the MGARP promoter that is activated by endogenous or exogenous Sp1 in a dominant manner. Collectively, our findings suggest a Sp1 regulatory mechanism in MGARP transcriptional regulation, with ERα functioning cooperatively with Sp1.

## Materials and Methods

### Plasmids and Reagents

The bioinformatics analysis was carried out as described in Text S1. The bacterial artificial chromosome clone bearing the MGARP gene (BAC, RP11-468C4) was purchased from Invitrogen (Carlsbad, CA, US). The MGARP promoter (−3 kb) was amplified by PCR using the following primers: 5′ GCT AAG CTT ATT CCA CAG AGA GGC TGA GAG-3′ and 5′-TAT GGA TCC GGA CTT TCT TAA CTG CTT GCC-3′. The cloned MGARP promoter was digested with *Bam*HI and *Hin*dIII and inserted into the pGL3-basic (Promega, Madison, WI, USA) and pDsRed-Express-1 (Clontech, Palo Alto, CA, USA) vectors to generate the pGL3-MGARP, pGL3-(−3 kb) and pDsRed-MGARP reporters. The MGARP promoter from the pGL3-(−3 kb) plasmid was digested by *Xba*I and *Hin*dIII to obtain two Sp1 binding regions that were subcloned into pGL3-basic and pDsRed-Express-1 to form corresponding reporters. The region (−150/0 bp) encompassing both GC-boxes was named Box1&2; the region (−150/−40 bp) containing the far proximal GC-Boxes was named Box1; and the region (−39/0 bp) containing the near proximal GC-Boxes was named Box2. To obtain the promoter regions with or without an Sp1-binding site, more oligos were synthesized and used for amplification (Table S1) to generate the indicated reporter plasmids. The plasmid containing the deletion of Box1 (DEL1) was named pGL3-DEL1; the plasmid containing the deletion of Box 2 (DEL2) was named pGL3-DEL2; and the plasmid containing the deletion of both BOX1 and BOX2 (DEL 1+2) was named pGL3-DEL1&2. The reporter clones were verified by sequencing. The two GC-Boxes were also isolated and cloned separately or jointly to generate the indicated reporters, with a *Sal*I site that exists between two Sp1 binding motifs used for the separation of the two Boxes. As the result, the following reporters were generated: pGL3-Box1&2, pGL3-Box1 and pGL3-Box2. For knockdown of Sp1, four short hairpin oligos targeting the 630 position (upper strand sequence: 5′-GAT CCA CCA ACA GAT TAT CAC AAA TTC AAG AGA TTT GTG ATA ATC TGT TGG TTT TTT TGG AAA-3′; lower strand sequence: 5′-AGC TTT TCC AAA AAA ACC AAC AGA TTA TCA CAA ATC TCT TGA ATT TGT GAT AAT CTG TTG GTG-3′) and 1722 position (upper strand sequence: 5′-GAT CCG TAC ATG ATG ACA CAG CAG GTT CAA GAG ACC TGC TGT GTC ATC ATG TAT TTT TTG GAA A-3; lower strand sequence: 5′-AGC TTT TCC AAA AAA TAC ATG ATG ACA CAG CAG GTC TCT TGA ACC TGC TGT GTC ATC ATG TAC G-3′) of the Sp1 gene were synthesized and cloned into pSilencer, generating two shRNA expression plasmids, 630-RNAi and 1722-RNAi, respectively. Their effectiveness was tested by western blotting (Text S2). The Sp1 expression plasmid was a kind gift from Dr. Jon Horowitz (Department of Molecular Biomedical Sciences, North Carolina State University, College of Veterinary Medicine) and was sent to us with the ERα plasmid by Dr. Shaoyong Chen (BIDMC, Harvard Medical School, USA). The Sp1 antibody was purchased from Millipore (Upstate, MA, USA).

### Cell Culture, Transfection, Luciferase (Luc) Assay and Red Fluorescence Protein Detection

HEK-293T cells were obtained from the Cell Resource Center (IBMS, CAMS/PUMC, BJ, China) and were grown in DMEM (Hyclone, Logan, UT, USA) supplemented with 10% fetal bovine serum (FBS) (ExCell Biology, SH, China) and penicillin/streptomycin. The reporters were transfected, as indicated, into HEK-293T cells using Vigofect reagent (Vigorous Biotechnology, BJ, China) according to the manufacturer’s protocol. After 6 hours, the medium was replaced with DMEM containing 10% FBS and antibiotics. 72 hours post transfection, cells were harvested for Luc assay using the Luc assay system (Vigorous Biotechnology, BJ, China), and the activity of Firefly luciferase values were normalized to that of the Renilla luciferase.

### Semiquantitative RT-PCR Analysis

Total RNA from HEK-293T cells transfected with scramble or specific RNAi (6 µg/well for 6-well plate) was extracted using TRIzol (Invitrogen, Carlsbad, California, US). Reverse transcription was performed using 1 µg of total RNA with the RNA PCR Kit of AMV Ver.3.0 (Takara, DL, China). Primer sequences for MGARP transcripts were 5′-ATGTATCTCCGCAGGGCGGT-3′ and 5′-CCCTTTGAGCCGAAGCAGC-3′ and for Sp1 transcripts, they were 5′-CTAAGTCCTAGCTAAGTATCAGG-3′ and 5′-AGAGGATGACAGACTTAGGAAGG-3′. The thermal cycling profile for the PCR reaction was the following: 95°C for 30 s, 58°C for 30 s and 72°C for 1 min for 25 PCR cycles. Products were resolved with 1.5% agarose gels.

### Electrophoretic Mobility Gel Shift Assay (EMSA)

HEK-293T and Y-1 mouse adrenocortical cells (Y1) (Cell bank of Chinese Academy of Sciences, SH, China) were cultured in 60 mm plates in DMEM or RPMI 1640 containing 10% FBS, transfected with the Sp1 plasmid, and then grown for additional 48 hours. The nuclear proteins were extracted and the total protein concentration of nuclear extracts was determined using the BCA assay (Pierce, Rockford, IL, USA). The values obtained were subsequently used for normalization. The oligonucleotides corresponding to the proximal GC boxes in the MGARP promoter were synthesized, annealed and 3′ end-labeled using the Biotin Kit (Invitrogen, Carlsbad, CA, US). LightShift Chemiluminescent EMSA kit (Thermo, Rockford, IL, USA) was used with 20 µl binding reaction system containing 1X Binding Buffer, 50 ng/µl of Poly (dI•dC) and crude nuclear extracts of HEK-293T and Y1 cells. 4 pmol of unlabeled competitor oligonucleotides, mutated unlabeled competitor oligonucleotides and 20 fmol of labeled probe were applied and incubated on ice for 10 min. The sequence of biotin labeled probe corresponding to the Sp1 binding region is 5′-TCCCGGGGGCCG- CGGAGGCGGGG CTGGAT-3′ (BOX1). The Sp1 antibody (07-645, Upstate, MA, USA) reaction with nuclear proteins was carried out for 10 min before the probe reaction was initiated. DNA-protein complexes were loaded onto a 5% SDS-PAGE gel for blotting. Analysis of ERα interaction with the MGARP promoter was carried out similarly.

### ChIP Assay

ChIP was performed on HEK-293T cells using the Chromatin Immunoprecipitation Assay Kit (Upstate, MA, USA) according to the manufacturer’s instructions. Briefly, 4×10^6^ cells were cross-linked by incubation with formaldehyde at a final concentration of 1% for 10 min at room temperature. The reaction was then quenched by glycine. The cells were subsequently washed twice with cold PBS, lysed in 400 µl SDS lysis buffer containing protease inhibitor cocktail II and subjected to sonication (7 × 10 sec). Then soluble chromatin was incubated with IgG and agarose for 1hour to pre-clear the chromatin. Immunoprecipitations were carried out by incubating with anti-Sp1 antibody (07-645, Upstate, MA, USA), anti-RNA polymerase II (Pol II) antibody (05-623B, Upstate, MA, USA) or negative control IgG (sc-2027, Santa Cruz, CA, USA) overnight at 4°C with rotation. Agarose beads were then added for a one-hour incubation at 4°C, followed by washing. The crosslink was reversed by heating overnight at 65°C, followed by treatments with RNase A for 30 min at 37°C and proteinase K for 2 hours at 45°C. The DNA was purified by phenol extraction and ethanol precipitation. The PCR reactions were carried out for 33 cycles with the following parameters: 94°C for 20 s, 56°C for 30 s, and 72°C for 30 s. The sequences of primers spanning the proximal GC-boxes (BOX1&2) of the MGARP gene promoter were sense, 5′-AGGAGTTACATTCAGTGGTACAGAA-3′; and antisense, 5′-CCTTCCACAGAGAGGCTGAGAGCCT-3′. The expected amplicon was 257 bp, and the PCR product was resolved by gel electrophoresis on 2.0% agarose.

### Statistical Analysis

Multiple groups (at least 3 replicates for each test) of data for parallel reporter studies were analyzed based on the ratio of the firefly luciferase value to that of the renilla luciferase, using the SigmaPlot software for one Way ANOVA to generate specific P values. A value of P<0.05 was considered to be significant.

## Results

### Defining the Promoter Region of the Human MGARP Gene

**Figure 1:**
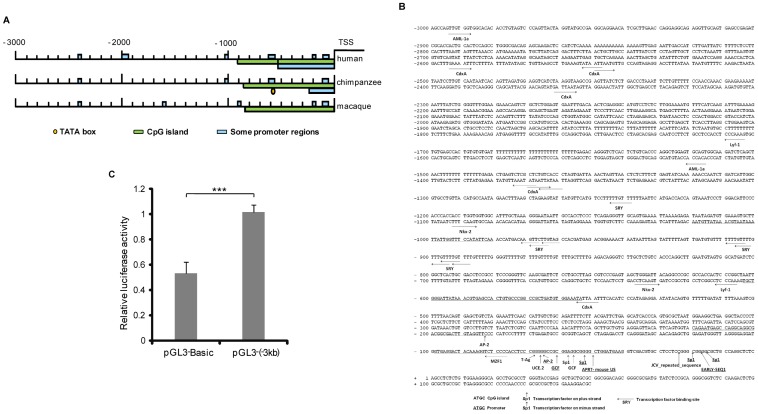
Bioinformatics analysis of the MGARP promoter. A. Promoter analysis comparing the −3 kb upstream region of the MGARP gene between three species (Homo sapiens, Pan troglodytes and Macaca mulatta). B. A summarized display of the −3 kb upstream region in the human MGARP promoter with predicted promoter and transcription factor (TF) binding sites. C. Luciferase (Luc) reporters driven by the 3 Kb MGARP promoter (pGL3-(−3 kb)) and the promoter-less control (pGL3-Basic) were transfected into HEK-293T cells and analysis was carried out as described in Materials and Methods. *** represents p<0.001 and ^#^represents p>0.05 (no significant difference).

After performing a BLAST search of the NCBI database, the MGARP gene was found to be located on the minus strand of the *Homo sapiens* chromosome 4 genomic contig **(**The accession number of this genomic contig in GenBank is NW-922217.**)** Additionally, a −10 kb region upstream of the TSS was submitted for prediction of promoter properties. A typical CpG island, but no obvious TATA box, was identified in the proximal −3 kb region of the first exon (Figure 1A). For further definition, we compared the −3 kb promoter regions of three species and, as shown in Figure 1A, there are some key features of the promoter those are highly conserved among *Homo sapiens* (Human), *Pan troglodytes* (Chimpanzee) *and Macaca mulatta* (Macaque). Particularly, these promoters share marked enrichment of a CpG island in the proximal region (Figure 1A). Further analysis of the −3 kb human MGARP promoter region for binding elements revealed putative binding sites for the following 13 transcription factors: AM-1a, CdxA, Lyf-1, SRY, Nkx-2, Ap-2, MZF1, T-Ag, UCE.2, GCF, Sp1, APRT and EARLY-SEQ1 (Figure 1B). Among them, Sp1 binding sites were identified at high frequencies and were particularly enriched in the proximal region that is highly conserved and GC-rich (Figure 1B). The two potential Sp1 binding motifs, (∼−20 bp to ∼−60 bp) and (0 ∼−20 bp), were located in close proximity to the TSS, each containing two Sp1 binding elements (Figure 1B). These two motifs were identified as two GC-boxes, a far proximal GC-box (∼−20 bp to ∼−60 bp; Box1) and a near proximal GC-box (0 ∼−20 bp; Box2).

To verify the activity of the putative MGARP promoter, the 3 kb DNA region upstream of the first exon of MGARP (−3 kb) was cloned into the pGL3-basic and pDsRed-Express-1 vectors to generate reporters for Luc analysis (pGL3-MGARP) and red fluorescence reporter (pDsRed-MGARP), respectively. The results demonstrated that the cloned MGARP promoter had basal activity in both reporter systems (Figure 1C, Figure S1).

### Sp1 Enhances MGARP Promoter Activity and Mediates MGARP Transcription

**Figure 2:**
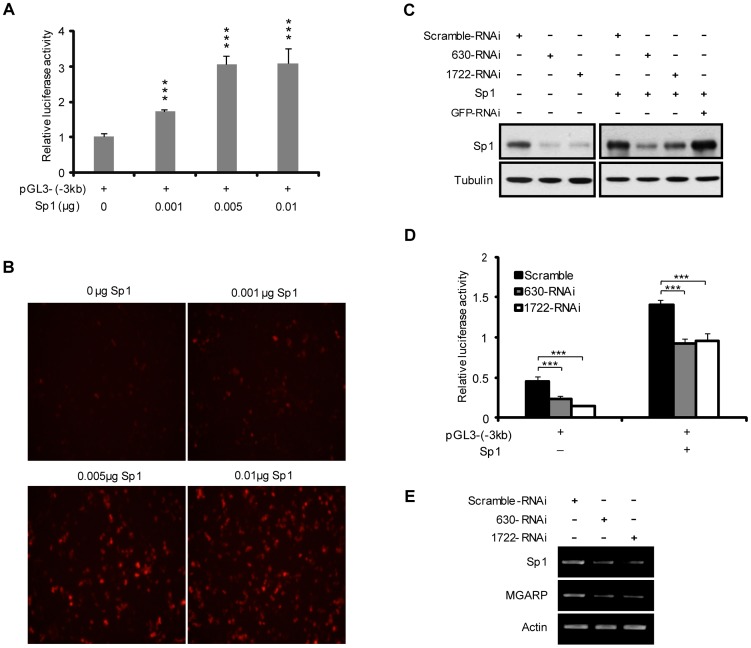
MGARP promoter activation is mediated by Sp1. A. Luc assay shows that Sp1 mediates the MGARP promoter activity in a dose-dependent manner. HEK-293T cells were co-transfected with pGL3-(−3 kb) and the increasing doses of Sp1 plasmids for Luc assay. B. Similarly, the pDsRed-MGARP(−3 kb) reporter and several doses of Sp1 plasmids were co-transfected into HEK-293T cells to examine the expression of red fluorescent protein at 72 hours post transfection. C. Western blotting shows that knockdown of Sp1 with Sp1-specific RNAi (630-RNAi and 1722-RNAi) reduces the expression of both endogenous and exogenous Sp1. The scramble-RNAi and RNAi targeting GFP were used as control. D. Luc assays to determine the effect of Sp1 knockdown on MGARP promoter activity. *** represents p<0.001 (E) Semiquantitative RT-PCR analysis confirms that knockdown of Sp1 reduces the MGARP gene expression. Similarly, HEK-293T cells were transfected with control and Sp1-specific RNAi and harvested for semiquantitative RT-PCR analysis of MGARP messages expression.

The above analysis indicated that Sp1 is a candidate transcriptional factor for MGARP gene expression. To clarify this finding, the pGL3-MGARP and pDsRed-MGARP reporters driven by the −3 kb MGARP-promoter were each cotransfected into HEK-293T cells, with increasing doses of Sp1 expression plasmids. As shown in Figure 2A and 2B, both Luc and fluorescence protein assay results showed that co-expression of Sp1 significantly increased the MGARP promoter activity in a dose-dependent manner. To further confirm the involvement of Sp1 in MGARP promoter activation, we designed two oligo pairs that specifically targeted the Sp1 gene and generated two shRNA expression vectors, 630-RNAi and 1722-RNAi. As shown by western blotting, both 630-RNAi and 1722-RNAi could effectively reduce the expression of endogenous and exogenous Sp1, as compared to the control (pSilencer-scramble plasmid) (Figure 2C). Significantly, cotransfection of either 630-RNAi or 1722-RNAi with the pGL3-MGARP reporter led to a remarkable decrease in luciferase activity, in both the absence or presence of co-expressed Sp1 (Figure 2D). Moreover, RT-PCR results showed that down-regulation of Sp1 by specific RNAi could reduce the endogenous MGARP gene expression (Figure 2E). These findings demonstrate that Sp1 is a critical transactivator for transcriptional activation of the MGARP promoter.

### Sp1 Activates the MGARP Promoter via Two GC-rich Motifs

**Figure 3:**
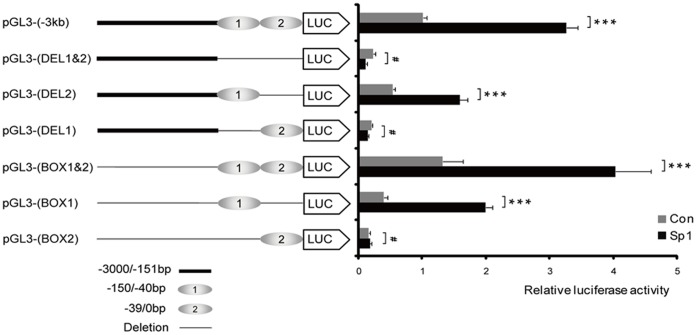
GC-box1 plays a major role in MGARP promoter activation and both GC-boxes are required for full transactivation. The Luc reporters driven by the full-length MGARP promoter (−3 kb) were transfected into HEK-293T cells, as compared to various promoter truncates either missing the GC-Boxes or expressing the GC-Boxes alone, without or with co-transfection of Sp1 plasmids (**10 ng**) as indicated. Luc activity was examined at 72 hours post transfection. *** represents p<0.001 and ^#^represents p>0.05 (no significant difference).

As detailed above, two GC-boxes were identified in the proximal MGARP promoter centered at ∼0 bp and ∼−60 bp, respectively. Each GC-box is clustered with two Sp1 binding elements. To clarify which GC rich motif is essential for Sp1-mediated regulation, several reporter constructs were generated with or without the GC-boxes (Figure 3). Results of the reporter assay indicated that the following promoters carried basal activity: pGL3-MGARP (−3 kb), pGL3-Box1&2, pGL3-Box1 and pGL3-DEL2; and that the luciferase activity derived from these promoters was significantly enhanced by co-expressed Sp1. However, the following reporters suggested transcriptional silence in the absence and presence of exogenous Sp1: pGL3-Box2, pGL3-DEL1&2 and pGL3-DEL1 (Figure 3). These findings indicate that GC-Box1 plays a dominant role to mediate Sp1-dependent transactivation of the MGARP promoter, and it requires both GC-Boxes to achieve full transcriptional activity. Additionally, the pGL3-Box1&2 promoter produced comparable (or slightly higher) luciferase activity when compared to the full-length pGL3-MGARP promoter (pGL3-(−3 kb)) (Figure 3), suggesting that Sp1 is the predominant transcriptional activator for the −3 kb proximal promoter region.

As a complementary approach, a similar test was carried out with co-expressed Sp1 and pDsRed-MGARP promoter (−3 kb), pDsRed-Box1&2, pDsRed-Box1 or pDsRed-Box2 reporters. The intensity of the red fluorescence showed a similar pattern of these promoters’ activities as compared to that of the Luc assay, in the absence and presence of co-expressed Sp1 (Figure S2).

Together, these findings indicate that substantial activation of the MGARP promoter critically depends on Sp1 and the proximal 150-bp region (−150/0 bp) that contains two GC-rich boxes, and that a synergistic interaction between the two Sp1 binding motifs is required for effective promoter activation.

### Sp1 Binds to the GC Boxes of the MGARP Promoter

**Figure 4:**
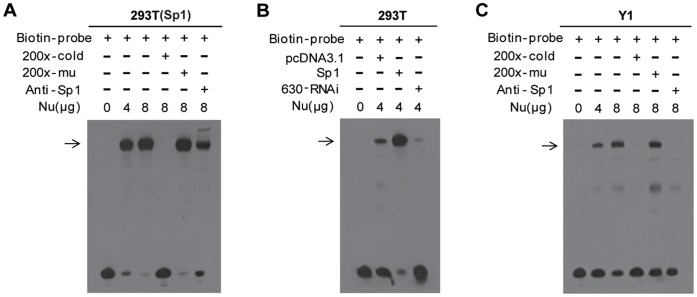
EMSA test indicates that Sp1 directly binds to the GC-boxes of the MGARP promoter. For the EMSA analysis, nuclear extracts (Nu) from HEK-293T or Y1 cells were incubated with Biotin-labeled oligonucleotides (Biotin-probe) spanning the GC-rich region (BOX1) of the MGARP promoter (−3 kb). Competition reactions were performed with 200X of unlabeled cold competitor (cold), 200X of mutated-labeled competitors (mu) or Sp1 antibody (2 µg). The following cell lines were used: non-transfected or Sp1-overexpressed HEK-293T cells (A), non-transfected, Sp1-overexpressed, or 630-RNAi transfected HEK-293T cells (B), and Y1 cells (C).

Next, we performed an EMSA to examine whether these GC boxes mediated the interaction of Sp1 with the MGARP promoter DNA backbone. Biotin-labeled short DNA oligos corresponding to Box1 were synthesized and annealed. Nuclear extracts from Sp1-overexpressed HEK-293T cells were incubated with the probe or the plain buffer as a control. As shown in Figure 4A, a shifted band was observed in the presence, but not the absence, of nuclear extracts, and the intensity of the band was associated with the concentrations of the extracts (Lane 2 and 3 in Figure 4A). Significantly, the shifted bands were eliminated when incubated with 200-fold excess unlabeled probe, but the mutated-unlabeled probe had no effect, indicating the specificity of Sp1 binding to the GC boxes of the MGARP promoter (Lane 4 and 5 in Figure 4A). At the same time, we attempted to super-shift the band by adding Sp1 specific antibody. After addition of the antibody to the reaction mixture, a super-shifted band was produced, and the amount of the corresponding shifted band was reduced (Lane 6 in Figure 4A).

Similarly, we performed an additional EMSA using HEK-293T cells subjected to Sp1-overexpression or RNAi-mediated Sp1 down-regulation. The results indicated that the endogenous Sp1 in HEK-293T cells could bind to the GC-boxes (control), overexpression of Sp1 markedly enhanced the intensity of the shifted band, and knockdown of Sp1 substantially reduced the binding, suggesting that this shifted band was Sp1-mediated (Figure 4B).

Since the HEK-293T cells were reported to have a relationship to neurons [27], and MGARP was demonstrated to be expressed in neurons and Y1 cells [4], [7], we examined and compared the expression of Sp1 and MGARP in HEK-293T and Y1 cells by Western blot. The results indicated that that both HEK-293T and Y1 cell could express endogenous Sp1 and MGARP proteins (Figure S3). The HEK-293T cells expressed more Sp1 and less MGARP while Y1 cells expressed less Sp1 and more MGARP proteins. To verify the above findings in an independent cellular system, Y1 cells were used because they express abundant MGARP protein and may contain a substantial amount of competent cofactors of Sp1 molecules that assist Sp1 in regulation of MGARP transcription. As shown in Figure 4C, a similar observation in binding was made by EMSA using Y1 cell nuclear extract, indicating that the endogenous Sp1 proteins can effectively bind the GC boxes of the MGARP promoter in different kinds of cell lines. However, with the addition of the antibody to the reaction mixture of Y1 cell nuclear extract, the super-shifted band disappeared (Figure 4C). This may indicate that an interaction of Sp1 with this antibody changed the molecular behaviors of Sp1, either by structurally altering the Sp1 protein, or by blocking the accessibility of the oligos. In any case, the test with the antibody indicated that the shifted bands specifically depend on Sp1.

**Figure 5:**
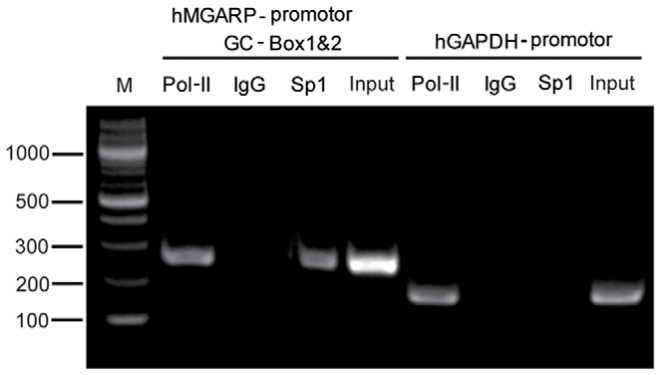
ChIP analysis indicates that Sp1 binds to MGARP promoter *in vivo*. ChIP was performed as described in the Materials and Methods. HEK-293T cells and antibodies for RNA polymerase II (Pol II) and Sp1 were used, with IgG as control. The immunoprecipitated chromatin was amplified by PCR with primers specific for the GC-rich region (BOX1&2) of the MGARP promoter (−3 kb), with GAPDH locus as control. M: DNA Marker.

To further validate the binding between Sp1 and GC-Boxes *in vivo*, we performed Chromatin Immunoprecipitation (ChIP) assay using HEK-293T cells. The results demonstrated that Sp1specifically bound to the GC-box locus on the endogenous MGARP promoter, but not to the control GAPDH locus (Figure 5). Together, our results suggest that Sp1 proteins directly bind to the proximal GC-rich region of the MGARP promoter.

### Sp1 and ER Synergistically Stimulate MGARP Promoter Activity

**Figure 6:**
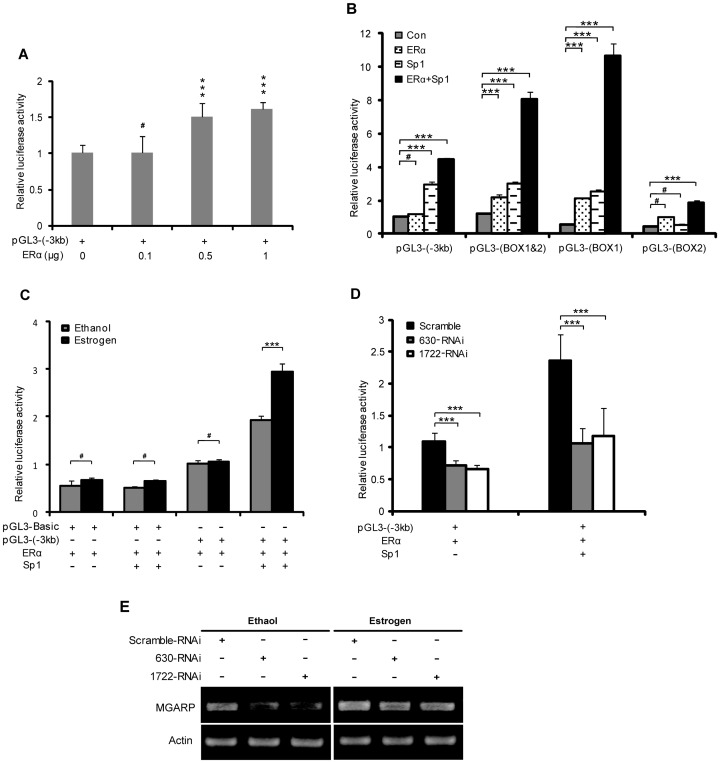
ERα up-regulates the transcription of the MGARP promoter and acts in synergy with Sp1 to activate MGARP transcriptional activity. A. pGL3-(−3 kb) reporter and different doses of ERα expression plasmid were co-transfected into HEK-293T cells to determine the dose-dependent manner of ERα in regulating the MGARP promoter by luciferase assay. B. The functional synergy between Sp1 and ERα was determined by cotransfection of the full-length MGARP promoter (−3 kb) or various promoter truncates with or without Sp1 plasmids for Luc assay as indicated. C. The synergystic transactivation activity of ERα and Sp1 under the stimulation of estrogens. The HEK-293T cells were treated with or without 10 nM of estradiol (E2) for 24 hours post transfection of pGL3-(−3 kb) and ERα. Subsequently, the Luc assay was performed at 72 hours post transfection. D. Knockdown of Sp1 diminishes the activation function of ERα on MGARP promoter. HEK-293T cells were co-transfected with pGL3-(−3 kb) reporter and ERα, together with Sp1-specific RNAi (630-RNAi or 1722-RNAi) or control RNAi, in the absence or presence of exogenous Sp1. *** represents p<0.001 and ^#^ represents p>0.05 (no significant difference). E. RT-PCR shows that down-regulation of Sp1 with Sp1-specific RNAi (630-RNAi or 1722-RNAi) results in a reduction in endogenous MGARP mRNA expression in HEK-293T cells when stimulated by 10 nM E2. The cells were treated with or without 10 nM E2 for 24 hours post transfection and total RNA was harvested at 72 hours post transfection for semiquantitative RT-PCR analysis.

It was reported that ERα interacts with Sp1 and they act synergistically to activate downstream genes [19], [28]. Considering that MGARP protein expression can be up-regulated by estrogens [5], we reasoned that ERα might be able to regulate the transcription of MGARP or coordinate with Sp1 in the activation of the MGARP promoter. This hypothesis was first tested by co-transfecting the MGARP promoter (pGL3-(−3 kb)) reporters with increasing concentrations of the ERα expression plasmids. The Luc assay results demonstrated that ERα could dose-dependently enhance MGARP transcriptional activity, indicating that this −3 kb upstream region may either contain non-classic ER-binding site(s) or engage with ERα-interacting transactivator(s), including endogenous Sp1 (Figure 6A). Importantly, co-expression of Sp1 with ERα can further increase ER-induced reporter activity, demonstrating significant synergistic effects on the MGARP promoters (Figure 6B). In addition, the synergistic effect was different for distinct regions of the MGARP promoter, with the promoters restricted to the GC Box1&2 and Box1 producing the most greatest synergy, further supporting that it is primarily mediated by Sp1 (Figure 6B). Since ERα can be activated by its natural ligand estrogen, we further studied the transactivation activity of ERα under the stimulation of estrogen. Our results indicated that estrogens could modestly enhance the transactivation activity of ERα on the MGARP promoter and markedly enhance the promoter activity in the presence of exogenous Sp1, while minimal effects were recorded on the control vector (Figure 6C). In contrast, in both the absence and presence of exogenous Sp1, knockdown of Sp1 significantly reduced the activation function of ERα on the MGARP promoter (Figure 6D). Furthermore, in ERα**-**transfected HEK-293T cells, estrogens could increase endogenous MGARP expression, while down-regulation of Sp1 led to a reduction in endogenous MGARP mRNA expression, in the absence and presence of estrogens (Figure 6E). Together, these findings demonstrate that Sp1 and ERα up-regulate MGARP promoter activity in a synergistic manner and that ERα may act as a co-activator for Sp1 to regulate MGARP promoter activity.

## Discussion

Gene transcription in eukaryotic organisms depends on the interplay between transcription factors and regulatory elements in promoters. Transcription is regulated by chromatin-interacting factors, which bind to their specific DNA recognition sequences [29]. Sp1 is a general transcription factor driving gene expression in early development [30], [31], containing a zinc finger motif that mediates binding to DNA with the consensus sequence 5′-(G/T)GGGCGG(G/A)(G/A)(C/T)-3′ (GC box element). We demonstrated that the region spanning -150 to 0 bp of the MGARP promoter fragment has basic promoter properties and contains multiple Sp1 binding sites that converge into two GC-Boxes. The enrichment of Sp1 binding motifs would potentially allow for the fine-tuning of transcription by this factor. Indeed, using a reporter assay we found that the MGARP promoter could be stimulated by Sp1 in a dose-dependent manner, suggesting that Sp1 functions as a limiting factor. In addition, integration of each GC-Box into basic reporters resulted in minimally active transcription and combining two GC-Boxes resulted in full activation of the promoter, indicating a synergistic mechanism between these two motifs. The findings that each individual GC-Box carries Sp1-activated promoter function and that a −150 bp proximal region is responsible for a significant part of MGARP promoter activity demonstrate that Sp1 is a dominant transactivator for MGARP expression. Comparing these two specific GC-rich Boxes, we propose that Box1 plays a major role in Sp1 transcriptional activity and that Box2 works cooperatively with Box1 to achieve full transactivation.

Our previous study showed that MGARP is highly expressed in the ovary, testis, retina and adrenal gland tissues, and its expression is under the regulation of the HPG axis [5]. MGARP has also been shown to be up-regulated by estrogens and its expression level correlates with the level of estrogens in the ovary during the estrous cycle [5]. These findings imply that MGARP functions in steroidogenesis and that MGARP is modulated by steroids [5]. In our computational promoter analysis, we did not identify classic ERα binding element(s) in the −3 kb proximal region; however, there still exists a possibility for direct ERα engagement with the proximal or distal promoter via non-classical binding site(s). In any case, here we demonstrate that ERα can stimulate the MGARP promoter in a dose-dependent manner. We further determined that ERα co-expression can stimulate Sp1-mediated promoter activation and this synergy can be further enhanced by estrogens. This suggests the existence of cross-talk between ERα and Sp1 at this gene locus, consistent with the reported findings that estrogens can enhance ERα-Sp1 interactions [32], [33]. Moreover, the critical dependence of ERα stimulatory effects on the GC Boxes and Sp1 indicated that Sp1 plays a dominant role in this synergistic interaction. The magnitude of ERα stimulatory effects on the MGARP promoter may depend on the ratio and sufficiency of each of the components in the systems, the availability of Sp1 and estrogens, and the structural composition of the promoter. The isolated mini MGARP promoter (tandem Sp1 elements) has a higher basal activity and more substantial response to ERα than the full-length −3 kb promoter, indicating that there are other factors in the −3 kb promoter contributing to the transcriptional regulation and the effects of ERα. Together, these findings suggested that ERα may potentiate MGARP transcription by serving as a co-activator for Sp1.

Sp1 is an abundant nuclear protein in most cells, but Sp1 protein levels showed marked differences during development and varied in different cell types [14], [34]. Sp1 protein expression was highest in the spermatids of sexually mature animals [34]. Sp1 knockout embryos are retarded in development, show a broad range of abnormalities, and die around day 11 of gestation [21]. As a classical nuclear and steroid receptor, ERα has profound implications in reproductive tract development and neuronal and vascular function [35]. Adult ER knockout mice exhibit several abnormalities and deficiencies, including phenotypic syndromes that result in infertility in both sexes [36]. More importantly, it has been well**-**documented that Sp1 and ERα synergistically regulate down-stream gene expression [32], [37]–[39], and their physical interaction enhances Sp1-DNA binding [33]. Our present data demonstrate that estrogen and ERα synergistically enhanced the MGARP promoter activation by Sp1. These findings provide explanations to our reported observations that MGARP was highly enriched in steroidogenic tissues and the neuronal/visual system and that estrogens up-regulate MGARP expression [4], [5], [7]. The physiological defects that resulted from targeted disruption of the ER gene [36] may be mediated, at least in part, by the deficiency in ER-Sp1 complex formation and reduction in MGARP expression that may cause aberrant steroid hormone synthesis, ultimately leading to diverse animal abnormalities, especially infertility.

Although reporter analysis of these two GC boxes indicated a prominent role for the 150-bp proximal region in MGARP promoter activity, it remains possible that the flanking regions also contribute to the transcription of the endogenous MGARP gene. As shown in Figure 3, the −3 kb promoter responds to the transfected Sp1 less than the minimal GC-Boxes (−150 bp), indicating that additional factors (or suppressors) may exist in this region and are involved in regulating the MGARP promoter activity. By bioinformatics analysis, we identified multiple chromatin-associated transcriptional factors that potentially bind to the promoter in the flanking regions of the GC-Boxes. The contribution of these proteins to MGARP transcription needs to be verified and may be important. For example, the −3 kb promoter region contains multiple binding motifs for SRY, an androgen receptor co-repressor [40]. SRY is a sex-determining gene on the Y chromosome, and mutations in this gene can cause abnormal sex development [41]–[43]. The regulation of MGARP by SRY, if validated, would suggest a pathway by which the androgen receptor modulates MGARP expression and in turn, MGARP may also mediate the biological and physiological effects of SRY. Together, our data suggest that the proximal promoter is Sp1-centered and the dependence of Sp1 for endogenous MGARP expression indicates that Sp1 is one major transactivator for the MGARP promoter, and additional regulatory factors may participate in regulation of the whole gene promoter.

In summary, we defined Sp1 as a major MGARP transactivator in the proximal MGARP promoter and identified two GC boxes in this region that mediate the transactivation of MGARP gene by Sp1. Sp1-driven transcription can be further enhanced by ERα, suggesting that the functional synergy between ER and Sp1 mediates the stimulating effect that estrogen was shown to have on MGARP transcription, providing a molecular mechanism for MGARP transcriptional regulation by steroids.

## Supporting Information

Figure S1 **Detection of basal activity of the MGARP promoter by red fluorescence.**
(DOCX)

Figure S2 **Analysis of different truncated MGARP promoter activity by red fluorescent protein.**
(DOCX)

Figure S3 **Endogenous expression of MGARP in HEK-293T cells and Y1 cells.**
(DOCX)

Table S1 Primers for constructing different deletion reporter vectors.
(DOCX)

Text S1 **Bioinformatics analysis.**
(DOCX)

Text S2 **Western blot assay.**
(DOCX)

## References

[1] Hennebold JD, Tanaka M, Saito J, Hanson BR, Adashi EY (2000) Ovary-selective genes I: the generation and characterization of an ovary-selective complementary deoxyribonucleic acid library. Endocrinology 141: 2725–2734. 10919256 10.1210/endo.141.8.7627

[2] Kinouchi R, Kinouchi T, Hamamoto T, Saito T, Tavares A, et al (2006) Distribution of CESP-1 protein in the corneal endothelium and other tissues. Invest Ophthalmol Vis Sci 47: 1397–1403. 16565373 10.1167/iovs.05-0602

[3] Qi S, Wang Y, Zhou M, Ge Y, Yan Y, et al (2010) A mitochondria-localized glutamic acid-rich protein (MGARP/OSAP) is highly expressed in retina that exhibits a large area of intrinsic disorder. Mol Biol Rep 38(5): 2869–2877. 20107910 10.1007/s11033-010-9948-x

[4] Matsumoto T, Minegishi K, Ishimoto H, Tanaka M, Hennebold JD, et al (2009) Expression of ovary-specific acidic protein in steroidogenic tissues: a possible role in steroidogenesis. Endocrinology 150: 3353–3359. 19325000 10.1210/en.2008-1584 PMC2703556

[5] Zhou M, Wang Y, Qi S, Wang J, Zhang S (2011) The Expression of a Mitochondria-Localized Glutamic Acid-Rich Protein (MGARP/OSAP) Is Under the Regulation of the HPG Axis. Endocrinology 152(6): 2311–2320. 21447634 10.1210/en.2011-0050

[6] Li Y, Rempe DA (2010) During hypoxia, HUMMR joins the mitochondrial dance. Cell Cycle 9: 50–57. 20016276 10.4161/cc.9.1.10318

[7] Li Y, Lim S, Hoffman D, Aspenstrom P, Federoff HJ, et al (2009) HUMMR, a hypoxia- and HIF-1alpha-inducible protein, alters mitochondrial distribution and transport. J Cell Biol 185: 1065–1081. 19528298 10.1083/jcb.200811033 PMC2711615

[8] McEwen BS (2010) Stress, sex, and neural adaptation to a changing environment: mechanisms of neuronal remodeling. Ann N Y Acad Sci 1204 Suppl: E38–59 20840167 10.1111/j.1749-6632.2010.05568.x PMC2946089

[9] Gorodeski GI (2005) Aging and estrogen effects on transcervical-transvaginal epithelial permeability. J Clin Endocrinol Metab 90: 345–351. 15483084 10.1210/jc.2004-1223

[10] Gibbs RB (1994) Estrogen and nerve growth factor-related systems in brain. Effects on basal forebrain cholinergic neurons and implications for learning and memory processes and aging. Ann N Y Acad Sci 743: 165–196, discussion 197–169. 10.1111/j.1749-6632.1994.tb55792.x 7802412

[11] Bergman MD, Karelus K, Felicio LS, Nelson JF (1989) Differential effects of aging on estrogen receptor dynamics in hypothalamus, pituitary and uterus of the C57BL/6J mouse. J Steroid Biochem 33: 1027–1033. 2615349 10.1016/0022-4731(89)90405-6

[12] Weihua Z, Makela S, Andersson LC, Salmi S, Saji S, et al (2001) A role for estrogen receptor beta in the regulation of growth of the ventral prostate. Proc Natl Acad Sci U S A 98: 6330–6335. 11371645 10.1073/pnas.111150898 PMC33468

[13] Pettersson K, Gustafsson JA (2001) Role of estrogen receptor beta in estrogen action. Annu Rev Physiol 63: 165–192. 11181953 10.1146/annurev.physiol.63.1.165

[14] Dynan WS, Tjian R (1983) Isolation of transcription factors that discriminate between different promoters recognized by RNA polymerase II. Cell 32: 669–680. 6187469 10.1016/0092-8674(83)90053-3

[15] Jin W, Chen Y, Di GH, Miron P, Hou YF, et al (2008) Estrogen receptor (ER) beta or p53 attenuates ERalpha-mediated transcriptional activation on the BRCA2 promoter. J Biol Chem 283: 29671–29680. 18765668 10.1074/jbc.M802785200 PMC2662053

[16] Sugimoto H, Okamura K, Sugimoto S, Satou M, Hattori T, et al (2005) Sp1 is a co-activator with Ets-1, and Net is an important repressor of the transcription of CTP:phosphocholine cytidylyltransferase alpha. J Biol Chem 280: 40857–40866. 16157598 10.1074/jbc.M503578200

[17] Hu P, Kinyamu HK, Wang L, Martin J, Archer TK, et al (2008) Estrogen induces estrogen-related receptor alpha gene expression and chromatin structural changes in estrogen receptor (ER)-positive and ER-negative breast cancer cells. J Biol Chem 283: 6752–6763. 18174157 10.1074/jbc.M705937200

[18] Wang W, Dong L, Saville B, Safe S (1999) Transcriptional activation of E2F1 gene expression by 17beta-estradiol in MCF-7 cells is regulated by NF-Y-Sp1/estrogen receptor interactions. Mol Endocrinol 13: 1373–1387. 10446910 10.1210/mend.13.8.0323

[19] Krishnan V, Wang X, Safe S (1994) Estrogen receptor-Sp1 complexes mediate estrogen-induced cathepsin D gene expression in MCF-7 human breast cancer cells. J Biol Chem 269: 15912–15917. 8195246

[20] Li R, Knight JD, Jackson SP, Tjian R, Botchan MR (1991) Direct interaction between Sp1 and the BPV enhancer E2 protein mediates synergistic activation of transcription. Cell 65: 493–505. 1850324 10.1016/0092-8674(91)90467-d

[21] Marin M, Karis A, Visser P, Grosveld F, Philipsen S (1997) Transcription factor Sp1 is essential for early embryonic development but dispensable for cell growth and differentiation. Cell 89: 619–628. 9160753 10.1016/s0092-8674(00)80243-3

[22] Martino A, Holmes JH IV, Lord JD, Moon JJ, Nelson BH (2001) Stat5 and Sp1 regulate transcription of the cyclin D2 gene in response to IL-2. J Immunol 166: 1723–1729. 11160217 10.4049/jimmunol.166.3.1723

[23] Goldhar AS, Duan R, Ginsburg E, Vonderhaar BK (2011) Progesterone induces expression of the prolactin receptor gene through cooperative action of Sp1 and C/EBP. Mol Cell Endocrinol 335: 148–157. 21238538 10.1016/j.mce.2011.01.004 PMC3045478

[24] Suzuki A, Sanda N, Miyawaki Y, Fujimori Y, Yamada T, et al (2010) Down-regulation of PROS1 gene expression by 17beta-estradiol via estrogen receptor alpha (ERalpha)-Sp1 interaction recruiting receptor-interacting protein 140 and the corepressor-HDAC3 complex. J Biol Chem 285: 13444–13453. 20200160 10.1074/jbc.M109.062430 PMC2859504

[25] Li D, Mitchell D, Luo J, Yi Z, Cho SG, et al (2007) Estrogen regulates KiSS1 gene expression through estrogen receptor alpha and SP protein complexes. Endocrinology 148: 4821–4828. 17656465 10.1210/en.2007-0154

[26] Safe S, Kim K (2008) Non-classical genomic estrogen receptor (ER)/specificity protein and ER/activating protein-1 signaling pathways. J Mol Endocrinol 41: 263–275. 18772268 10.1677/JME-08-0103 PMC2582054

[27] Shaw G, Morse S, Ararat M, Graham FL (2002) Preferential transformation of human neuronal cells by human adenoviruses and the origin of HEK 293 cells. FASEB J 16: 869–871. 11967234 10.1096/fj.01-0995fje

[28] Danesh SM, Kundu P, Lu R, Stefani E, Toro L (2011) Distinct transcriptional regulation of human large conductance voltage- and calcium-activated K+ channel gene (hSlo1) by activated estrogen receptor alpha and c-Src. J Biol Chem 286(36): 31064–31071. 21757754 10.1074/jbc.M111.235457 PMC3173123

[29] Ptashne M (1988) How eukaryotic transcriptional activators work. Nature 335: 683–689. 3050531 10.1038/335683a0

[30] Courey AJ, Holtzman DA, Jackson SP, Tjian R (1989) Synergistic activation by the glutamine-rich domains of human transcription factor Sp1. Cell 59: 827–836. 2512012 10.1016/0092-8674(89)90606-5

[31] Courey AJ, Tjian R (1988) Analysis of Sp1 in vivo reveals multiple transcriptional domains, including a novel glutamine-rich activation motif. Cell 55: 887–898. 3142690 10.1016/0092-8674(88)90144-4

[32] Khan S, Barhoumi R, Burghardt R, Liu S, Kim K, et al (2006) Molecular mechanism of inhibitory aryl hydrocarbon receptor-estrogen receptor/Sp1 cross talk in breast cancer cells. Mol Endocrinol 20: 2199–2214. 16675542 10.1210/me.2006-0100

[33] Porter W, Saville B, Hoivik D, Safe S (1997) Functional synergy between the transcription factor Sp1 and the estrogen receptor. Mol Endocrinol 11: 1569–1580. 9328340 10.1210/mend.11.11.9916

[34] Saffer JD, Jackson SP, Annarella MB (1991) Developmental expression of Sp1 in the mouse. Mol Cell Biol 11: 2189–2199. 2005904 10.1128/mcb.11.4.2189-2199.1991 PMC359911

[35] Couse JF, Korach KS (1999) Estrogen receptor null mice: what have we learned and where will they lead us? Endocr Rev 20: 358–417. 10368776 10.1210/edrv.20.3.0370

[36] Eddy EM, Washburn TF, Bunch DO, Goulding EH, Gladen BC, et al (1996) Targeted disruption of the estrogen receptor gene in male mice causes alteration of spermatogenesis and infertility. Endocrinology 137: 4796–4805. 8895349 10.1210/endo.137.11.8895349

[37] Fleming JG, Spencer TE, Safe SH, Bazer FW (2006) Estrogen regulates transcription of the ovine oxytocin receptor gene through GC-rich SP1 promoter elements. Endocrinology 147: 899–911. 16254027 10.1210/en.2005-1120

[38] Safe S, Kim K (2004) Nuclear receptor-mediated transactivation through interaction with Sp proteins. Prog Nucleic Acid Res Mol Biol 77: 1–36. 15196889 10.1016/S0079-6603(04)77001-4

[39] Khan S, Abdelrahim M, Samudio I, Safe S (2003) Estrogen receptor/Sp1 complexes are required forinduction of cad gene expression by 17beta-estradiol in breast cancer cells. Endocrinology 144: 2325–2335. 12746293 10.1210/en.2002-0149

[40] Yuan X, Lu ML, Li T, Balk SP (2001) SRY interacts with and negatively regulates androgen receptor transcriptional activity. J Biol Chem 276: 46647–46654. 11585838 10.1074/jbc.M108404200

[41] Wallis MC, Waters PD, Graves JA (2008) Sex determination in mammals–before and after the evolution of SRY. Cell Mol Life Sci 65: 3182–3195. 18581056 10.1007/s00018-008-8109-z PMC11131626

[42] Koopman P (1995) The molecular biology of SRY and its role in sex determination in mammals. Reprod Fertil Dev 7: 713–722. 8711208 10.1071/rd9950713

[43] Goodfellow PN, Lovell-Badge R (1993) SRY and sex determination in mammals. Annu Rev Genet 27: 71–92. 8122913 10.1146/annurev.ge.27.120193.000443

