# Supplementary material for: Mitochondria-Localized Glutamic Acid-Rich Protein (MGARP) Gene Transcription Is Regulated by Sp1
Source: PLoS One. 2012 Nov 27;7(11):e50053. doi: 10.1371/journal.pone.0050053 (PMC3507827; doi:10.1371/journal.pone.0050053)
Supplement: Figure S1 — Detection of basal activity of the MGARP promoter by red fluorescence. (DOCX) [file pone.0050053.s001.docx]

**Figure S1**


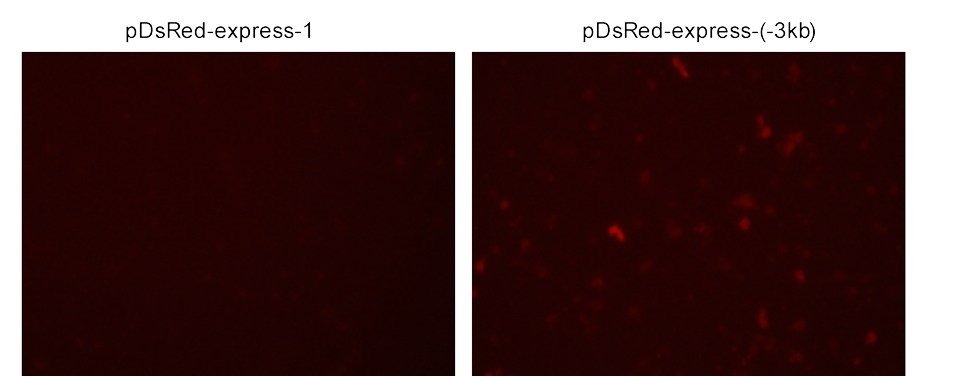


Figure S1. Detection of basal activity of the MGARP promoter by red fluorescence. HEK-293T cells were co-transfected with MGARP (-3 kb) – RFP and observed at 72 hr post transfection.
