# Supplementary material for: Mitochondria-Localized Glutamic Acid-Rich Protein (MGARP) Gene Transcription Is Regulated by Sp1
Source: PLoS One. 2012 Nov 27;7(11):e50053. doi: 10.1371/journal.pone.0050053 (PMC3507827; doi:10.1371/journal.pone.0050053)
Supplement: Figure S2 — Analysis of different truncated MGARP promoter activity by red fluorescent protein. (DOCX) [file pone.0050053.s002.docx]

**Figure S2**


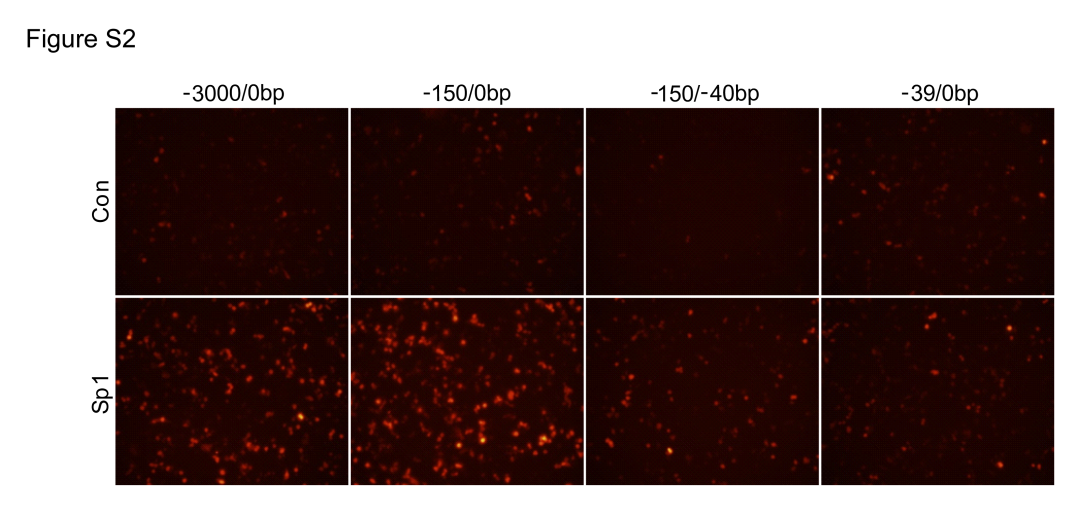


Figure S2. Analysis of different truncated MGARP promoter activity by red fluorescent protein. pDsRed reporters driven by the above MGARP promoters were transfected into HEK-293T cells with or without the Sp1 plasmids (10ng) for observation of red fluorescence.
