# Supplementary material for: Mitochondria-Localized Glutamic Acid-Rich Protein (MGARP) Gene Transcription Is Regulated by Sp1
Source: PLoS One. 2012 Nov 27;7(11):e50053. doi: 10.1371/journal.pone.0050053 (PMC3507827; doi:10.1371/journal.pone.0050053)
Supplement: Figure S3 — Endogenous expression of MGARP in HEK-293T cells and Y1 cells. (DOCX) [file pone.0050053.s003.docx]

**Figure S3**


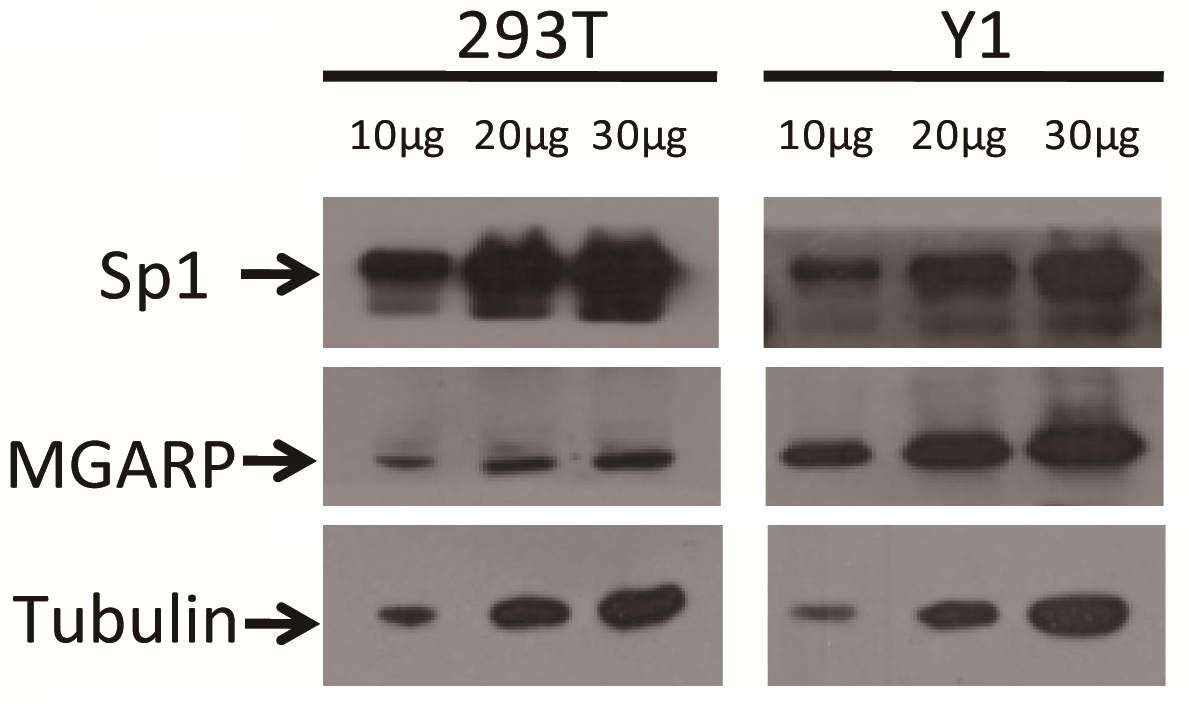


Figure S3. Endogenous expression of MGARP in HEK-293T cells and Y1 cells. The cells were harvested and lysed to release the proteins and then different dose of proteins were loaded on the gels and tested by Western blot. Tubulin from these two kinds of cells was used as control
