# Supplementary material for: Mitochondria-Localized Glutamic Acid-Rich Protein (MGARP) Gene Transcription Is Regulated by Sp1
Source: PLoS One. 2012 Nov 27;7(11):e50053. doi: 10.1371/journal.pone.0050053 (PMC3507827; doi:10.1371/journal.pone.0050053)
Supplement: Table S1 — Primers for constructing different deletion reporter vectors. (DOCX) [file pone.0050053.s004.docx]

**Table S1 Primers for constructing different deletion reporter vectors**

| **Primer names** | **Sequences** | **Enzyme site** |
| --- | --- | --- |
| MGRPP1 | 5'-TAT***GGTACC***GGACTTTCTTAACTGCTTGCC-3 | *Kpn* 1 |
| MGRPP1b | 5'-TAT***GGATCC***CTGACCGCGGCATCTCAGAAAA-3' | *Bam*H1 |
| MGRPP2b | 5'-TAT***GGATCC***TTCATCCAGCCCCGCCTCC-3' | *Bam*H1 |
| MGRPP3b | 5'-TAT***GGATCC***GAGAGGCTGAGAGCCTGGCAGCGCCCCGCCCGGAGGAGGCACGTCGACTGAGGTCTCTAGAGCTGACCG-3' | *Bam*H1 |

**Note:** For the deletion of Box1 (DEL1), two oligos, MGRPP1 and MAPP3b, were used to amplify the target fragment. The PCR product was digested by Kpn1+ BamH1 and ligated to the pGL3 vector by Kpn1+Bgl II to form a plasmid, named pGL3-DEL1. For the deletion of Box 2(DEL2), the target fragment was amplified by the oligos of MGRPP1+MGRPP2b and digested Kpn1+ BamH1 and ligated to the pGL3 vector by Kpn1+Bgl II to form a plasmid, named pGL3-DEL2. To clone the deletion of both BOX1 and BOX2 (DEL 1+2), the oligos of MGRPP1 and MAPP1b were used for PCR. The PCR product was digested by Kpn1+ BamH1 and ligated to the pGL3 vector by Kpn1+Bgl II to form a plasmid, named pGL3-DEL1+2.
