# Supplementary material for: Mitochondria-Localized Glutamic Acid-Rich Protein (MGARP) Gene Transcription Is Regulated by Sp1
Source: PLoS One. 2012 Nov 27;7(11):e50053. doi: 10.1371/journal.pone.0050053 (PMC3507827; doi:10.1371/journal.pone.0050053)
Supplement: Text S1 — Bioinformatics analysis. (DOCX) [file pone.0050053.s005.docx]

**Supporting information**

**Text S1**

**Bioinformatics analysis**

DNA sequences from a -100kb region upstream of the MGARP coding sequence were submitted for exon definition through FirstEF (http://rulai.cshl.edu/tools/FirstEF/). Further definitions of the transcription start sites (TSS) and the genomic structure of this gene were achieved by comparing various versions of cDNA sequences and expressed sequence tags (ESTs) in the NCBI database. An alignment was performed to compare highly conserved DNA sequences from *Homo sapiens, Pan Troglodytes and Macaca mulatta.* The -3kb upstream promoter of MGARP was predicted to contain converging elements and transcription factor (TF) prediction for this region was performed. The links used for the bioinformatics analysis are shown below:

*FirstEF:* http://rulai.cshl.edu/tools/FirstEF

*CpGProD:* http://pbil.univ-lyon1.fr/software/cpgprod_query.html

*Softberry–TSSW:* http://www.softberry.com/berry.phtml?topic=tssw&group=programs&subgroup=promoter

*Softberry–TSSG:* http://www.softberry.com/berry.phtml?topic=tssg&group=programs&subgroup=promoter

*WWW Promoter Scan:* http://www-bimas.cit.nih.gov/molbio/proscan/

*Promoter 2.0:* http://www.cbs.dtu.dk/services/Promoter/

*Softberry–CpGFinder:* http://www.softberry.com/berry.phtml?topic=cpgfinder&group=programs&subgroup=promoter

*NNPP:* http://www.fruitfly.org/seq_tools/promoter.html

*WebGene Server：*http://l25.itba.mi.cnr.it/cgi-bin/wwwcpg.pl

*GrailEX:* http://compbio.ornl.gov/grailexp/

*TESS:* http://www.cbil.upenn.edu/cgi-bin/tess/tess

*TFSEARCH:* <http://www.cbrc.jp/research/db/TFSEARCH.html>.
