# Supplementary material for: Mitochondria-Localized Glutamic Acid-Rich Protein (MGARP) Gene Transcription Is Regulated by Sp1
Source: PLoS One. 2012 Nov 27;7(11):e50053. doi: 10.1371/journal.pone.0050053 (PMC3507827; doi:10.1371/journal.pone.0050053)
Supplement: Text S2 — Western blot assay. (DOCX) [file pone.0050053.s006.docx]

**Western Blot Assay**

HEK-293T cells and Y1 cells were harvested when cell density reached 90% confluence and lysed by RIPA buffer. Protein concentration was measured using BCA protein assay (Thermo, Rockford, IL, USA). Different amount of protein (10µg, 20µg and 30 µg) was seperated on 10% SDS-PAGE and then transferred to nitrocellulose membrane by semi-dry transfer. The membranes were blocked with 5% powered milk in TBST for 1 hr at RT and the membranes were incubated with Sp1 antibody, MGARP antibody and TUBULIN antibody at 4ºC overnight. Membranes were washed for 3 times with 1XTBST and incubated with the secondary antibody linked with horseradish peroxidases for 60 min at RT. The membranes were developed with enhanced chemiluminescence and exposed to film.
